# Release of moth pheromone compounds from *Nicotiana benthamiana* upon transient expression of heterologous biosynthetic genes

**DOI:** 10.1186/s12915-022-01281-8

**Published:** 2022-03-31

**Authors:** Yi-Han Xia, Bao-Jian Ding, Shuang-Lin Dong, Hong-Lei Wang, Per Hofvander, Christer Löfstedt

**Affiliations:** 1 Department of Biology, Lund University, Sölvegatan 37, SE-22362 Lund, Sweden; 2 Department of Biology and Biological Engineering, Chalmers University of Technology, Kemivägen 4, SE-41296 Gothenburg, Sweden; 3 Education Ministry Key Laboratory of Integrated Management of Crop Diseases and Pests, College of Plant Protection, Nanjing Agricultural University, Nanjing, CN-210095 China; 4 Department of Plant Breeding, Swedish University of Agricultural Sciences, P.O. Box 101, SE-23053 Alnarp, Sweden

**Keywords:** Functional characterization, Fatty acyl desaturases, Fatty acyl reductase, Alcohol oxidation, Acetyltransferase, Heterologous expression systems, Pheromone-releasing plants

## Abstract

**Background:**

Using genetically modified plants as natural dispensers of insect pheromones may eventually become part of a novel strategy for integrated pest management.

**Results:**

In the present study, we first characterized essential functional genes for sex pheromone biosynthesis in the rice stem borer *Chilo suppressalis* (Walker) by heterologous expression in *Saccharomyces cerevisiae* and *Nicotiana benthamiana*, including two desaturase genes *CsupYPAQ* and *CsupKPSE* and a reductase gene *CsupFAR2*. Subsequently, we co-expressed *CsupYPAQ* and *CsupFAR2* together with the previously characterized moth desaturase *Atr∆11* in *N. benthamiana*. This resulted in the production of (*Z*)-11-hexadecenol together with (*Z*)-11-hexadecenal, the major pheromone component of *C. suppressalis*. Both compounds were collected from the transformed *N. benthamiana* headspace volatiles using solid-phase microextraction. We finally added the expression of a yeast acetyltransferase gene *ATF1* and could then confirm also (*Z*)-11-hexadecenyl acetate release from the plant.

**Conclusions:**

Our results pave the way for stable transformation of plants to be used as biological pheromone sources in different pest control strategies.

**Supplementary Information:**

The online version contains supplementary material available at 10.1186/s12915-022-01281-8.

## Background

Moths rely strongly on sex pheromones for mate communication. Synthetic pheromones have been used for monitoring, mass trapping, and mating disruption in integrated pest management (IPM) for several decades [1, 2] as environmentally friendly alternatives or complements to conventional insecticides. They are species-specific and non-toxic, and the risk of pests evolving resistance to their own pheromone is very low. Compared to current standard approaches to pheromone synthesis [3], the use of biological factories for pheromone production may have several advantages, allowing cost-efficient production of moderate to large quantities of pheromones with high purity and a minimum of waste. A series of proof-of-concept studies have clearly demonstrated the potential of producing moth pheromones in both plant and yeast factories [4–7] to replace conventionally produced pheromones in existing systems for pheromone-based pest control. However, under certain circumstances, it may be advantageous to cultivate plants that actually release the pheromone volatiles in the field rather than accumulate them for harvest. Such pheromone-releasing plants could hypothetically and depending on context either protect themselves against moth pests by causing mating disruption or be cultivated together with other harvest crops for the purpose of attraction or mating disruption.

Deciphering the molecular mechanism of pheromone biosynthesis in moth species can provide a functional gene pool for producing customized pheromones in heterologous systems. Approximately 75% of the identified moth pheromones belong to the so-called type I sex pheromones; C_10_–C_18_ alcohols, acetates, or aldehydes that are biosynthesized from palmitic and stearic acid by consecutive steps of fatty acyl desaturation, chain-shortening or chain-elongation, reduction, and final modification [8]. Specific enzymes are used for each catalytic step. Fatty acyl desaturases (FADs) are the first essential enzymes that introduce double bonds in specific positions of carbon chains with strict regioselectivity and stereoselectivity. Fatty acyl reductases (FARs), which are responsible for reducing fatty acyls to fatty alcohols, are essential for forming functional groups of the fatty acid derivatives [8]. The genes encoding the essential enzymes involved in pheromone fatty acyl desaturation and reduction have been functionally characterized in many moth species [9–25]. Characterization of FAD and FAR genes with different substrate and product specificities remains important for the production of tailored moth pheromones in heterologous systems.

The fatty alcohols may be actual pheromone components, or they may be further functionalized by the conversion of fatty alcohols into esters [26] or aldehydes [27–29]. Although in vivo labeling experiments have confirmed these biological reactions, no enzymes catalyzing the reactions in the pheromone glands have been identified and cloned from any insect species. In order to produce acetate pheromones in plants, Ding et al. [5] explored the possibility of using an acetyltransferase gene *EaDAcT* cloned from burning bush, *Euonymus alatus*. Transient expression resulted in the production of (*Z*)-11-hexadecenyl acetate (Z11-16:OAc), but the efficiency was low. Subsequently, Ding et al. [30] characterized a yeast acetyltransferase gene *ATF1* which efficiently acetylates insect pheromone alcohols into acetates in yeast. However, the activity of *ATF1* in plants remains to be explored.

The rice stem borer, *Chilo suppressalis* (Walker) (Lepidoptera: Crambidae), boring the stems of their host plants, is an infamous rice pest in East Asia, India, and Indonesia, causing great production reduction in rice crops [31]. In the 1970s and 1980s, the sex pheromone of female *C. suppressalis* was identified as a mixture of (*Z*)-11-hexadecenal (Z11-16:Ald), (*Z*)-13-octadecenal (Z13-18:Ald) [32, 33], and (*Z*)-9-hexadecenal (Z9-16:Ald) at the ratio of 100:13:11 [34]. In the present study, we functionally characterized several candidate genes likely to be involved in pheromone biosynthesis in *C. suppressalis* by heterologous expression in yeast and plant platforms, using the transcriptome data reported by Xia et al. [35]. We produced *N. benthamiana* genetically modified for expression of the functionally characterized *C. suppressalis* ∆11 desaturase *CsupYPAQ*, the fatty acyl reductase *CsupFAR2* and the yeast acetyltransferase *ATF1* that released a mixture of (*Z*)-11-hexadecenol (Z11-16:OH), Z11-16:OAc, and Z11-16:Ald. Our study contributes additional genes to the pool of key enzymes available for biotechnological production of moth pheromones, and more importantly, it is a significant step forward in the construction of genetically modified plants to be used as natural dispensers of insect pheromones as part of IPM strategies for pest control.

## Results

### *CsupYPAQ* and *CsupKPSE* are the functional FAD genes involved in pheromone biosynthesis in *Chilo suppressalis*

**Fig. 1:**
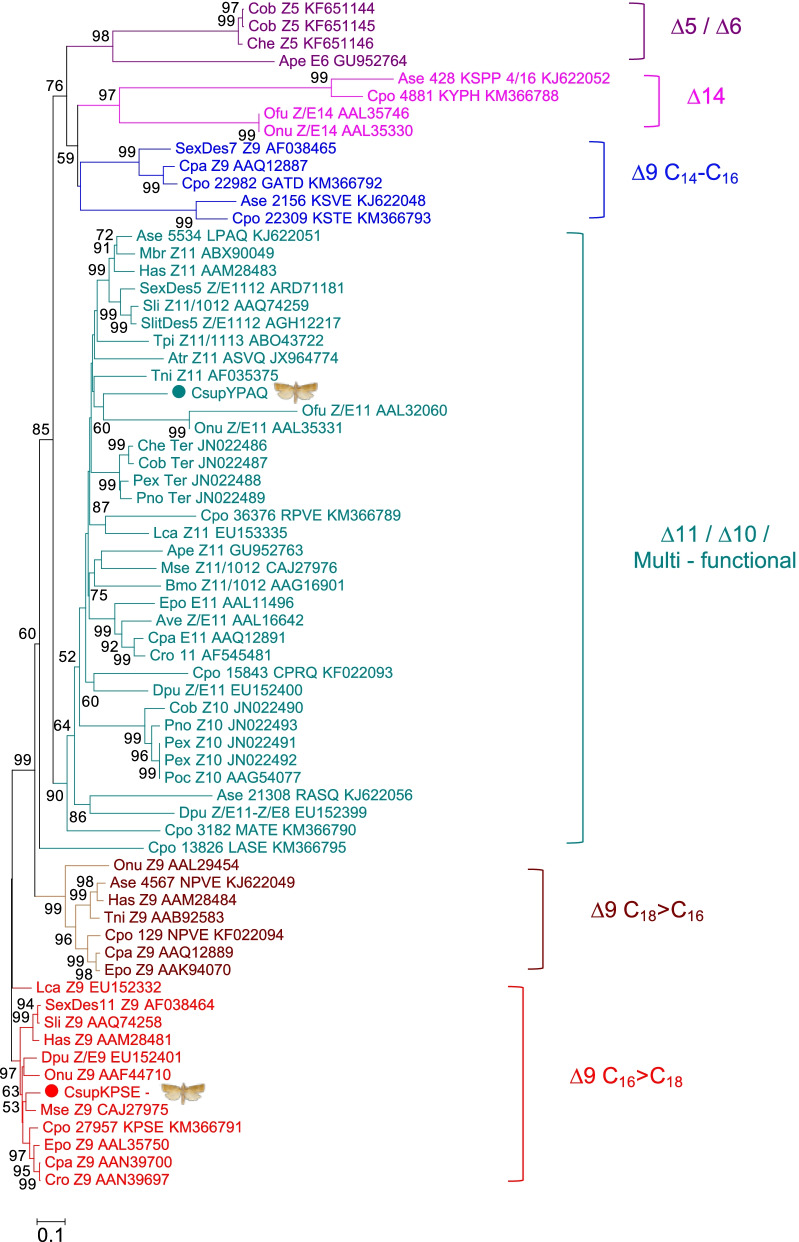
Phylogenetic tree of fatty acyl desaturases (FADs). The FAD tree was constructed by using amino acid sequences of lepidopteran FADs. The FADs functionally characterized in previous studies are classified into six subfamilies: ∆9 FAD subfamilies usually involved in normal fatty acid metabolism with preference for C_16_ (C_16_>C_18_) or C_18_ (C_18_>C_16_); the ∆11/∆10/multifunctional FAD subfamily; the ∆9 FAD subfamily with preference for C_14_-C_26_, involved in pheromone biosynthesis; and the ∆14 and ∆5/∆6 FAD subfamilies that introduce double bonds into unusual positions in saturated fatty acids. The predicted FAD genes *CsupYPAQ* and *CsupKPSE* from *C. suppressalis* are marked by green and red-filled circles, respectively. Values indicated at the nodes are bootstrap values based on 1000 replicates and bootstrap values <50% are not shown

The *C. suppressalis* FAD-like genes *CsupYPAQ* and *CsupKPSE* (following the nomenclature proposed by Knipple et al. [13] naming desaturase genes based on the composition of 4 amino acid residues at a signature motif) displayed 1038 nt and 1059 nt ORFs that translated into 346 and 353 aa-proteins, respectively. In a phylogenetic analysis of lepidopteran FADs, *CsupYPAQ* clustered into the ∆11/∆10/multifunctional FAD subfamily, whereas *CsupKPSE* fell into the ∆9 (C_16_>C_18_) FAD clade (Fig. 1).

**Fig. 2:**
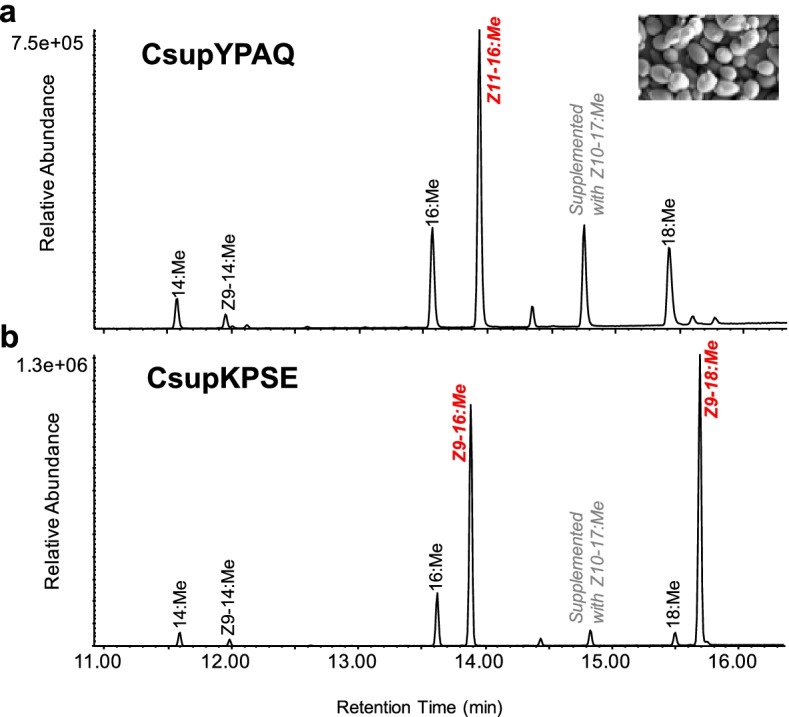
Heterologous expression of fatty acyl desaturase candidates from *Chilo suppressalis* in ∆*ole1*/*elo1 Saccharomyces cerevisiae*. GC/MS analysis of fatty acid methyl ester profiles of yeast expressing the *CsupYPAQ* or *CsupKPSE*. The compounds produced from the introduced desaturases are labeled in red italics. Methyl (*Z*)-10-heptadecenoate (Z10–17:Me) was supplemented as nutrition to all the incubations. Displayed chromatograms are representative examples of at least six replicates

**Fig. 3:**
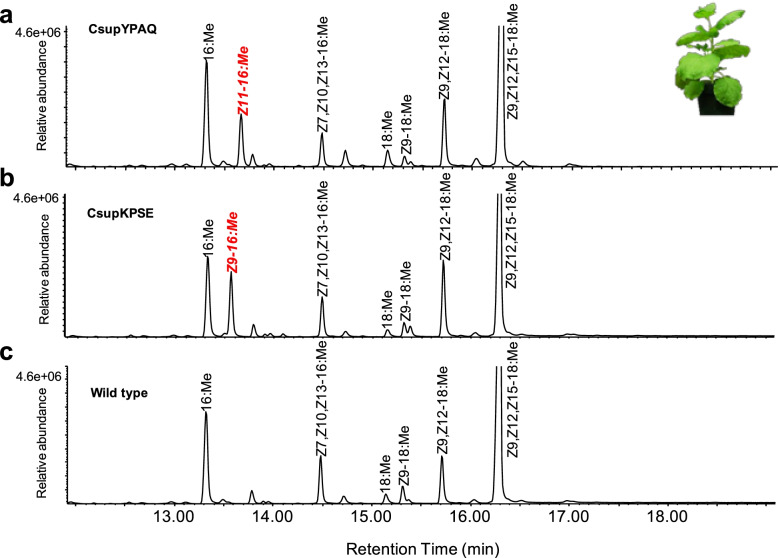
Heterologous expression of fatty acyl desaturase candidates from *Chilo suppressalis* in *Nicotiana benthamiana*. GC/MS analysis of fatty acid methyl ester profiles of **a** plant leaves expressing *CsupYPAQ*, **b** plant leaves expressing *CsupKPSE*, and **c** wild-type leaves. Native compounds from plants are labeled in black and the compounds produced from the introduced desaturases are labeled in red italics. Displayed chromatograms are representative examples of at least six replicates

The functional expression of *CsupYPAQ* and *CsupKPSE* indicated their involvement in pheromone biosynthesis. GC/MS analysis of yeast fatty acids showed that the yeast expressing *CsupYPAQ* produced a high amount of (*Z*)-11-hexadecenoic acid (Z11-16:acid) (Fig. 2a), while yeast expressing *CsupKPSE* produced high amounts of oleic acid (Z9-18:acid) and (*Z*)-9-hexadecenoic acid (Z9-16:acid) (Fig. 2b). Compared to the yeast expression results, *CsupYPAQ* showed a similar function in *N. benthamiana* (Fig. 3a). However, *CsupKPSE* expressed in *N. benthamiana* did not produce observable extra Z9-18:acid but only a very high amount of Z9-16:acid (Fig. 3b). In the wild type *N. benthamiana*, none of the monounsaturated potential pheromone precursors was produced (Fig. 3c).

### *CsupFAR2* is the FAR involved in pheromone biosynthesis in *Chilo suppressalis*

**Fig. 4:**
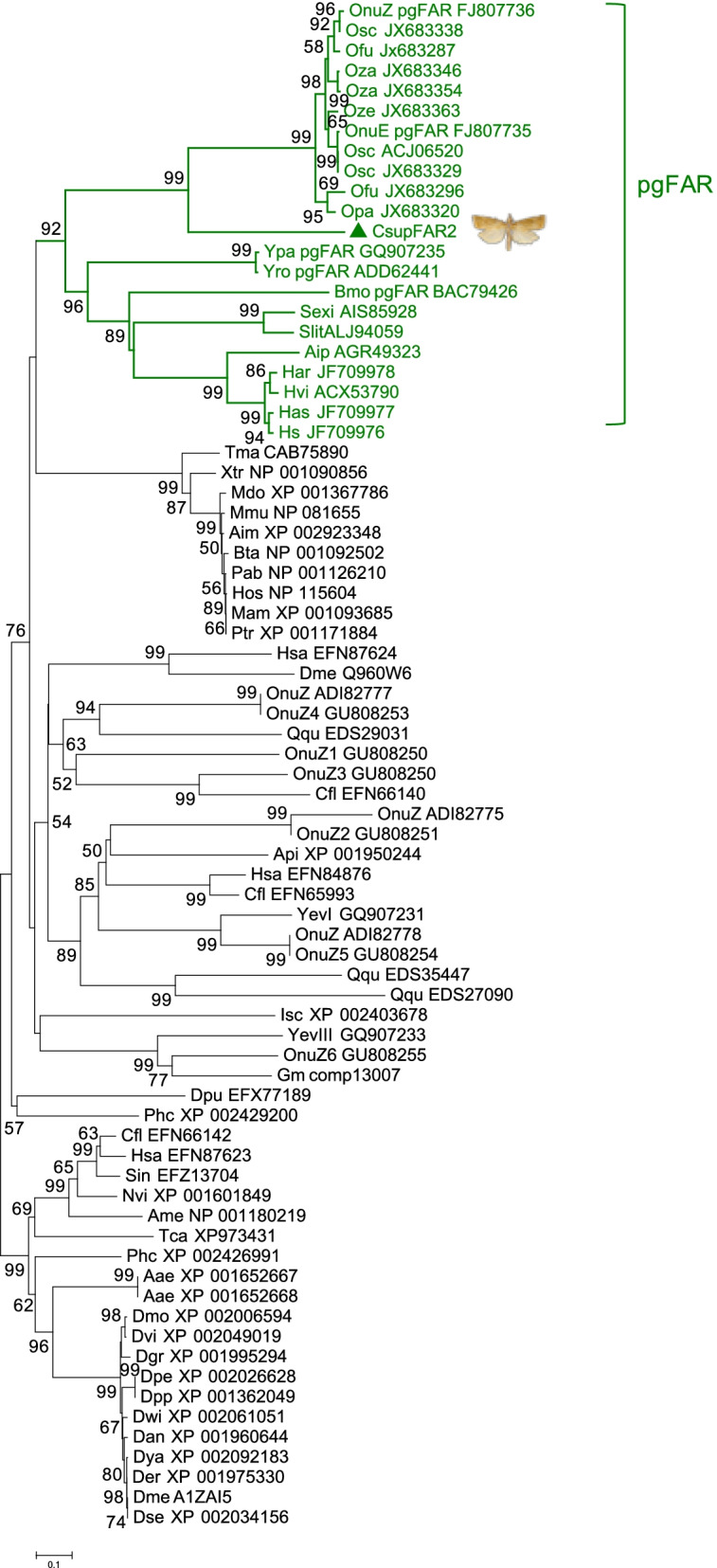
Phylogenetic tree of fatty acyl reductases (FARs). The tree is constructed from mammal, arthropod, and Lepidoptera FARs using amino acid sequences. The pgFAR clade, which contains previously functionally characterized FARs involved in moth pheromone biosynthesis, is shown in green and marked by a bracket. The predicted *C. suppressalis* fatty acyl reductase *CsupFAR2* is marked by a triangle. Values indicated at the nodes are bootstrap values based on 1000 replicates and bootstrap values <50% are not shown.

**Fig. 5:**
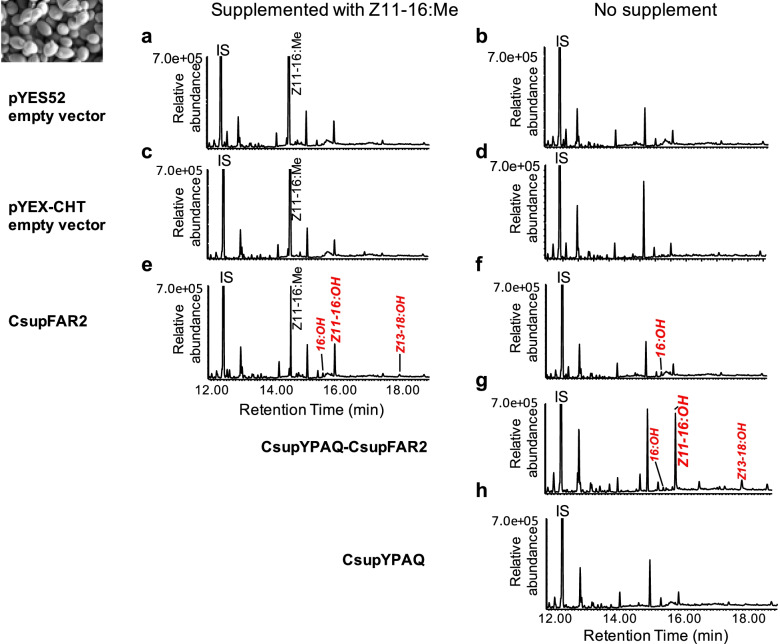
Heterologous expression of the fatty acyl reductase candidate gene *CsupFAR2* and the desaturase gene *CsupYPAQ* in *Saccharomyces cerevisiae INVSc* strain. *CsupFAR2* is constructed in the galactose inducible vector pYES52 and *CsupYPAQ*-*CsupFAR2* is constructed in the Cu^2+^ inducible vector pYEX-CHT. GC/MS analysis of fatty alcohol profiles of yeast harboring the **a** pYEX52 empty vector supplemented with Z11-16:Me, **b** pYES52 empty vector without supplement, **c** pYEX-CHT empty vector supplemented with Z11-16:Me, **d** pYEX-CHT empty vector without supplement, **e ***CsupFAR2* in pYES52 supplemented with Z11-16:Me, **f ***CsupFAR2* in pYES52 without supplement, **g ***CsupYPAQ-CsupFAR2*, and **h ***CsupYPAQ* in pYEX-CHT without supplement. Fatty alcohols produced from the introduced genes are shown in red and italics. Displayed chromatograms are representative examples of at least six replicates.

**Fig. 6:**
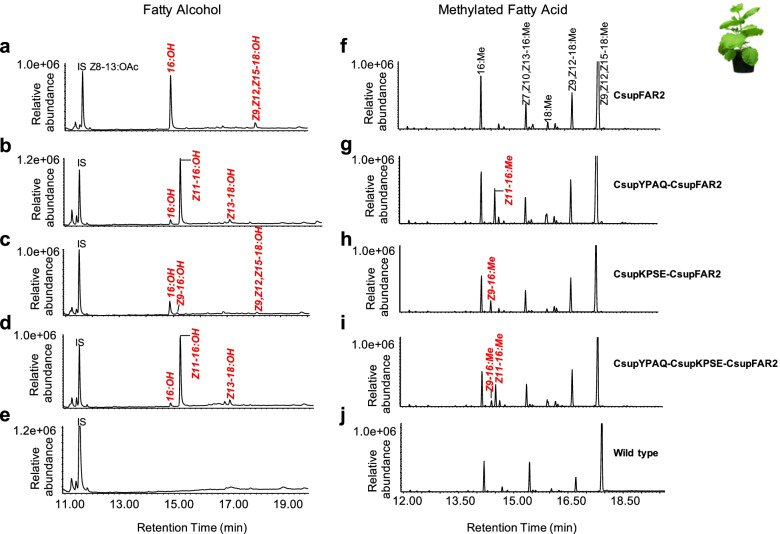
Heterologous expression of fatty acyl reductase candidate gene *CsupFAR2* and fatty acyl desaturase genes *CsupYPAQ* and *CsupKPSE* in *Nicotiana benthamiana*. GC/MS analysis of fatty alcohol (**a**–**e**) and corresponding fatty acid (**f**, **j**) profiles of plant leaves expressing the **a**, **f ***CsupFAR2*, **b**, **g ***CsupYPAQ-CsupFAR2*, **c**, **h ***CsupKPSE-CsupFAR2*, **d**, **i ***CsupYPAQ-CsupKPSE-CsupFAR2*, and wild type (**e** and **j**). Fatty alcohols produced by the introduced genes are shown in red. Eighteen micrograms of Z8-13:OAc per gram fresh leaf was added during the extraction as internal standard. Displayed chromatograms are representative examples of at least six replicates

The gene candidate *CsupFAR2* encompassed an ORF of 1404 nt, which corresponded to a 468 aa-protein. The phylogenetic analysis showed that *CsupFAR2* could be classified as a pgFAR (Fig. 4). The functional assays of *CsupFAR2* performed in both yeast and plant expression platforms demonstrated that it reduced the *C. suppressalis* pheromone precursors Z11-16:acid, Z9-16:acid, and (*Z*)-13-octadecenoic acid (Z13-18:acid) to the corresponding fatty alcohols (Figs. 5 and 6). A co-expression construct of *CsupYPAQ*-*CsupFAR2* was produced (ca. 3500 nt) with the *Gal1* promoter upstream of *CsupFAR2*. The yeast that was not expressing *CsupYPAQ* was supplemented with Methyl (*Z*)-11-hexadecenoate (Z11-16:Me). The yeast with the transformed empty vectors of Gateway adjusted pYES52 (Fig. 5a, b) and pYEX-CHT (Fig. 5c, d) did not produce fatty alcohol, while the yeast expressing *CsupFAR2* or *CsupYPAQ-CsupFAR2* produced Z11-16:OH, (*Z*)-13-octadecenol (Z13-18:OH), and palmityl alcohol (16:OH) (Fig. 5e–g). Compared to the yeast expressing only *CsupFAR2* and supplemented with Z11-16:Me, the yeast co-expressing *CsupYPAQ*-*CsupFAR2* (without supplement) produced a higher amount of target compounds (Fig. 5g). In the negative control, the yeast expressing *CsupYPAQ* only did not produce fatty alcohols (Fig. 5h). Besides Z11-16:OH, Z13-18:OH, and 16:OH, no other fatty alcohol species were detected in the yeast (Fig. 5).

In the *N. benthamiana* expression system, *CsupFAR2* was demonstrated to be active with a range of substrates including saturated, monounsaturated, and polyunsaturated fatty acids. The leaves expressing *CsupFAR2* reduced a high amount of 16:acid and a minor amount of α-linolenic acid (Z9,Z12,Z15-18:acid) to the corresponding 16:OH and linolenyl alcohol (Z9,Z12,Z15-18:OH) (Fig. 6a, f). However, it did not reduce other plant-derived fatty acids such as linoleic acid (Z9,Z12-18:acid), roughanic acid (Z7,Z10,Z13-16:acid), Z9-18:acid, or other saturated fatty acids than 16:acid in detectable amounts. When *CsupYPAQ* was co-introduced with *CsupFAR2*, the leaves produced plenty of Z11-16:OH. This gene combination resulted in a tenfold higher amount of Z11-16:OH than 16:OH and Z13-18:OH (Fig. 6b). When *CsupKPSE*-*CsupFAR2* was co-expressed in the plant, in addition to 16:OH and linolenyl alcohol, a minor amount of (*Z*)-9-hexadecenol (Z9-16:OH) was produced (less than 1/3 of 16:OH), similar to the proportion of the precursor Methyl (*Z*)-9-hexadecanoate (Z9-16:Me) relative to Methyl palmitate (16:Me) (Fig. 6c, h). The plant co-expressing multiple genes *CsupYPAQ*-*CsupKPSE*-*CsupFAR2* showed similar alcohol production compared to the plant expressing *CsupYPAQ*-*CsupFAR2* (Fig. 6b, d), but Z9-16:acid was only produced in the plants expressing the *CsupKPSE* (Fig. 6g, i). The wild-type plant did not produce any of the alcohols mentioned above (Fig. 6e).

### No functional alcohol oxidase genes characterized from *Chilo suppressalis*

A homology-based search yielded five fatty alcohol oxidase/dehydrogenase candidates (*CsupFAO_15570*, *CsupFAO_9572*, *CsupADH_10975*, *CsupADH_14583*, *CsupADH_17286*) from *C. suppressalis* (Table 1). The fatty alcohol oxidase (FAO) gene candidates encompassed ORFs around 1900 nt and had the highest amino acid identity of 26.5% between *CsupFAO_15570* and an *FAO* (XM_500864) from the yeast *Yarrowia lipolytica*. The alcohol dehydrogenase (ADH) gene candidates encompassed ORFs around 1100 nt and had the highest amino acid identity of 68.6% between *CsupADH_14583* and an *ADH* (NP_741507) from the nematode *Caenorhabditis elegans.*

**Table 1:** Fatty alcohol oxidase/dehydrogenase candidates from *Chilo suppressalis* tested in this study

Gene ID	Expression (RPKM)	ORF (bp)	BLAST search matching queries			
			Name	Acc. number	Species	Identity (%)
**CsupFAO_15570**	23.6	1896	Alcohol oxidase	XM_500864	*Yarrowia lipolytica*	26.5
			Alcohol oxidase	BAO74119	*Starmerella bombicola*	13.6
**CsupFAO_9572**	28.3	1893	Alcohol oxidase	XM_500864	*Yarrowia lipolytica*	26.1
			Alcohol oxidase	BAO74119	*Starmerella bombicola*	14.3
**CsupADH_10975**	8.8	1077	Alcohol dehydrogenase class3	NP_571924	*Danio rerio*	40.2
			Alcohol dehydrogenase class3	NP_741507	*Caenorhabditis elegans*	37.7
			Formaldehyde dehydrogenase	NP_524310	*Drosophila melanogaster*	34.9
			Formaldehyde dehydrogenase	XP_001693934	*Chlamydomonas reinhardtii*	34.5
			Alcohol dehydrogenase	NP_177837	*Arabidopsis thaliana*	30.9
			Alcohol dehydrogenase	NP_000660	*Homo sapiens*	26.3
			Alcohol dehydrogenase	NP_001075997	*Equus caballus*	25.1
			Bifunctional Alcohol dehydrogenase S288C	NP_010113	*Saccharomyces cerevisiae*	24.3
			Alcohol dehydrogenase	NP_031435	*Mus musculus*	24
**CsupADH_14583**	28.1	1131	Alcohol dehydrogenase class3	NP_571924	*Danio rerio*	77.7
			Formaldehyde dehydrogenase	NP_524310	*Drosophila melanogaster*	72.1
			Alcohol dehydrogenase class3	NP_741507	*Caenorhabditis elegans*	68.6
			Formaldehyde dehydrogenase	XP_001693934	*Chlamydomonas reinhardtii*	62.3
			Bifunctional Alcohol dehydrogenase S288C	NP_010113	*Saccharomyces cerevisiae*	60.2
			Alcohol dehydrogenase	NP_031435	*Mus musculus*	56.3
			Alcohol dehydrogenase	NP_000660	*Homo sapiens*	55.3
			Alcohol dehydrogenase	NP_001075997	*Equus caballus*	55.3
			Alcohol dehydrogenase	NP_177837	*Arabidopsis thaliana*	51.7
**CsupADH_17286**	284	1134	Formaldehyde dehydrogenase	XP_001693934	*Chlamydomonas reinhardtii*	21.9

We then individually co-expressed the five FAO and ADH-like genes and an additional ADH gene from *Helicoverpa zea HzeaADH7*, which was previously reported as an ADH-highly like gene with PG abundant expression in pheromone glands [36], in *N. benthamiana* leaves together with *CsupYPAQ* and *CsupFAR2* in various combinations (Additional file 1: Table S1).

**Fig. 7:**
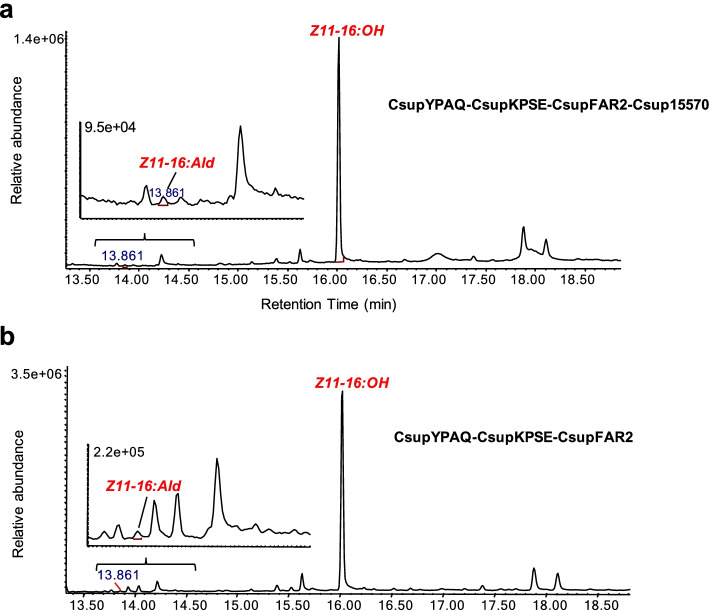
Heterologous expression of fatty alcohol oxidase candidate *Csup15570* with desaturase genes *CsupYPAQ* and *CsupKPSE* and reductase gene *CsupFAR2* from *Chilo suppressalis* in *Nicotiana benthamiana*. GC/MS analysis of Z11-16 fatty alcohol and aldehyde from plant leaves expressing **a** *CsupYPAQ*-*CsupKPSE-CsupFAR2*-Csup15570 and **b** *CsupYPAQ*-*CsupKPSE-CsupFAR2*. Fatty alcohol and aldehyde are shown in red. Displayed chromatograms are representative examples of at least six replicates

However, no results indicating the expected activity were obtained with any of the FAO/ADH gene candidates. In the GC/MS analysis of the leaf extracts, only a small peak of Z11-16:Ald was detected in the leaf samples when *CsupYPAQ*, *CsupKPSE*, *CsupFAR2*, and *Csup15570* were co-expressed in the plant (Fig. 7a) and the control leaf samples from co-expression of *CsupYPAQ*, *CsupKPSE*, and *CsupFAR2* also contained an albeit very small but still significant amount of Z11-16:Ald (Fig. 7b). This implied the existence of an endogenous plant activity that works on Z11-16:OH for producing Z11-16:Ald.

### Transiently genetically modified *Nicotiana benthamiana* releasing moth pheromones

**Fig. 8:**
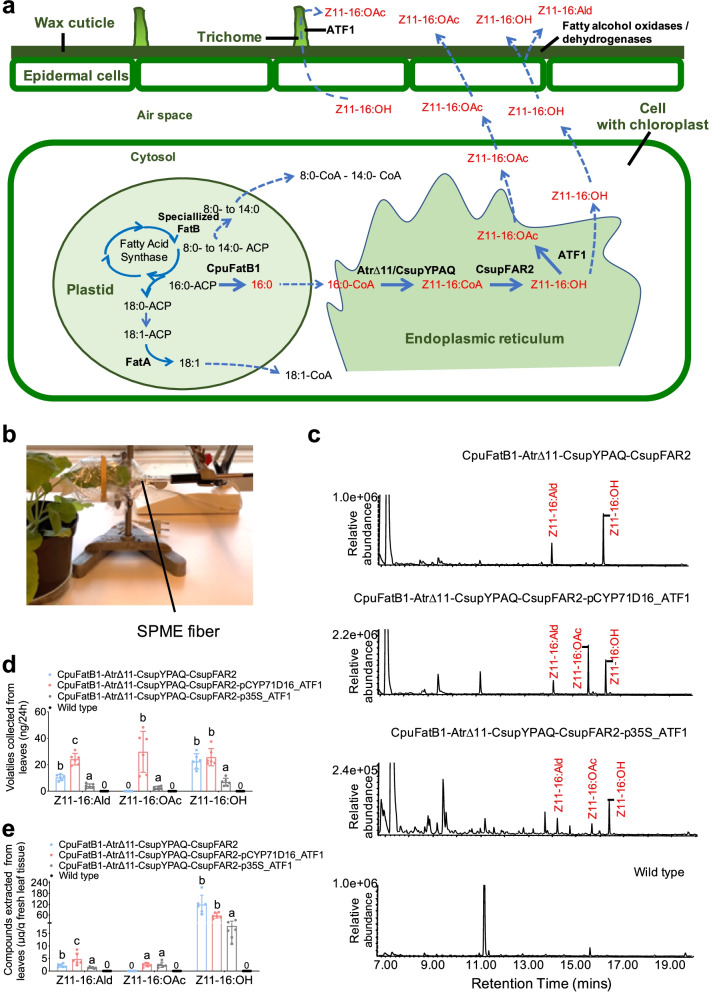
Rapid assembly of pheromone biosynthetic pathway in *Nicotiana benthamiana* for the production and release of moth sex pheromone components. The Cauliflower mosaic virus 35S promoter (*p35S*) and Octopine Synthase gene terminator (*tOCS*) have been used to regulate gene expression in plants. *ATF1* has also been controlled by trichome-specific promoter *pCYP71D16*. **a** Step-wise metabolic engineering strategy for leaf-based pheromone production of (*Z*)-11-hexadecenol, (*Z*)-11-hexadecenal, and (*Z*)-11-hexadecenyl acetate. **b** Solid-phase microextraction (SPME) of headspace volatiles from a genetically modified *N. benthamiana* leaf 4 days after infiltration. **c** GC-MS chromatograms (INNOWax column) of volatiles collected during 24 h from an infiltrated *N. benthamiana* leaf. **d** The amount of collected pheromone compounds from released volatiles. **e** The amount of pheromone compounds from leaf extracts. The error bars represent the standard deviation (SD). *N*=6. Bars with the same letters represent treatments that are not significantly different at the *P*=0.05 level (two-way ANOVA followed by *t*-tests)

The newly identified desaturase and reductase genes with desired properties were used together with two genes previously identified, to produce plants transiently genetically modified to release moth pheromone compounds. *Nicotiana benthamiana* plants infiltrated with the functional ∆11 desaturases gene *Atr∆11* and thioesterase gene *CpuFatB1*, and the newly identified desaturase and reductase genes *CsupYPAQ* and *CsupFAR2* (Fig. 8a), released a mixture of Z11-16:OH and Z11-16:Ald that could be collected as volatiles from the plant leaves (Fig. 8c, d). Upon co-expression of the acetyltransferase gene *ATF1*, Z11-16:OAc was also released together with Z11-16:OH and Z11-16:Ald. In addition, to explore the possibility of increasing the amount of moth pheromone compounds released from plant leaf, a *N. tabacum* trichome-specific promoter *pCYP71D16* was used to control the expression of acetyltransferase gene *ATF1*. Consequently, the plant released significantly higher amounts of Z11-16:OAc, as well as Z11-16:Ald and Z11-16:OH, compared to when *ATF1* expression was controlled by the constitutive promoter *p35S* (Fig. 8d). The accumulation of Z11-16:Ald and Z11-16:OH was also higher in the leaves when *ATF1* was controlled by *pCYP71D16*, while Z11-16:OAc accumulation in the leaves showed no significant difference between the two strategies (Fig. 8e).

## Discussion

Enabling plants to release heterologously produced moth pheromones is an essential prerequisite for developing an IPM strategy in which the “pheromone-releasing” plants can be used for mating disruption of pests or to selectively attract a specific pest insect. We successfully made *N. benthamiana* release the major pheromone compound, Z11-16:Ald, of *C. suppressalis* and the common pheromone compounds Z11-16:OH and Z11-16:OAc, by transient expression of the functionally characterized *C. suppressalis* ∆11 desaturase *CsupYPAQ*, the fatty acyl reductase *CsupFAR2,* and the yeast acetyltransferase gene *ATF1*. Furthermore, characterization of the highly active pheromone biosynthetic genes from *C. suppressalis* is an important addition to the genetic toolbox for constructing heterologous platforms to produce customized insect pheromones.

**Fig. 9:**
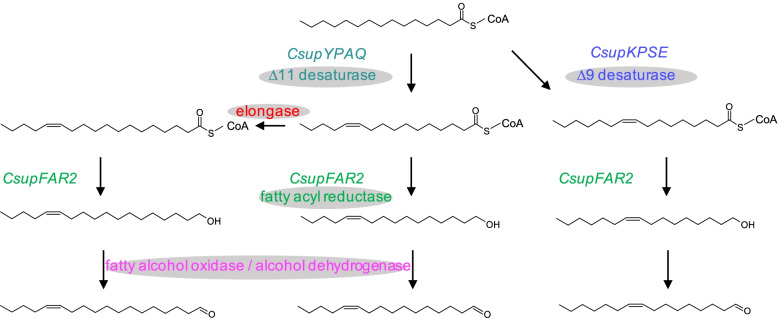
Biosynthetic pathways towards the sex pheromone of *Chilo suppressalis* and the key genes involved in each step

Understanding the mechanisms underpinning moth pheromone biosynthesis is considered the basis for developing biological factories for moth pheromone production. In the present study, we demonstrate the key roles of three genes in the biosynthesis of the *C. suppressalis* sex pheromone, i.e., a ∆11 desaturase *CsupYPAQ* that acts specifically on palmitic acid to produce Z11-16:acid; a ∆9 desaturase *CsupKPSE* producing Z9-16:acid, the precursor of the minor pheromone component from palmitic acid; and *CsupFAR2* that transforms the acid precursors into the corresponding fatty alcohols. The enzyme(s) transforming the alcohols into the final aldehyde pheromone components remains elusive (Fig. 9).

In insects, the acyl-CoA desaturase gene family has had a very dynamic evolutionary history [13, 37, 38]. As a result of gene duplication bursts, many opportunities have arisen for evolution to explore protein space and produce proteins with unique and novel functions. In phylogenetic analysis, *CsupYPAQ* clusters in the Lepidoptera-specific XXXQ/E clade (the proteins contain an XXXQ/E amino acid motif 13 amino acids in front of the conserved histidine box of the desaturase sequences) (Fig. 1), which is a lineage that is composed of pheromone biosynthetic desaturases with diverse specificities [13]. With the exception of the ∆9 desaturase from the tortricid species *Cydia pomonella* [39], the FADs clustering in this clade are ∆11/∆10 FADs but in some cases, they have additional functions and are then classified as multifunctional. For example, *Trichoplusia ni* and *Spodoptera exigua* from Noctuoidae using ∆11 [12] and ∆11/∆12 [22] desaturations, respectively; *Thaumetopoea pityocampa* from Notodontidae using ∆11/∆13 desaturations [20]; *Bombyx mori* from Bombycidae using ∆11/10,12 desaturations [40]; *Epiphyas postvittana*, *Choristoneura rosaceana*, and *Agryrotaenia velutinana* from Tortricidae using Δ11 desaturations [11, 14, 15], from the same family *Planotortrix octo* using Δ10 desaturation [10] (Fig. 1). Interestingly, even though *CsupYPAQ* clusters close to *OfuZ/E11* (*Ostrinia furnacalis*) and *OnuZ/E11* (*O. nubilalis*) from crambid species, which produce both *Z* and *E* isomers of ∆11-tetradecenoic acid [18], it specifically produces (*Z*)-11-hexadecenoic acid. In addition, *CsupYPAQ* shares 59.7% and 61.8% of amino acid identity to *SexiDes5* (*S. exigua*) and *SlitDes5* (*S. litura*), respectively, which are also both ∆11 FADs and have a very wide substrate preference, from 14 to 18 carbon chain length fatty acids [22]. All evidence at hand advocates for caution when predicting gene functions based on homology to currently characterized gene sequences, and functional testing is essential in terms of enhancing our mechanistic understanding of the relation between sequence and function of desaturases. Ding et al. [30] reported that one amino acid (258E) at the cytosolic carboxyl terminus of the protein is critical for the *Z* activity of the *C. rosaceana* FAD. The relative specificity of *CsupYPAQ* makes it an interesting candidate to further explore the correlation between the sequence and function of FADs by using site-directed mutagenesis and functional testing. Functional assays of *CsupYPAQ* showed similar results in the yeast and plant platforms but *CsupKPSE* expression did not yield oleic acid in *N. benthamiana* while it did in *S. cerevisiae*. Phylogenetically, *CsupKPSE* is clustered in the “∆9, C_16_>C_18_” clade; however, it showed C_18_>C_16_ function when oleic acid was absent in the *ole1*/*elo1* yeast.

Fatty acyl reductases are used for converting fatty acyls into their corresponding alcohols during moth pheromone biosynthesis. Sometimes, these FARs are referred to with the epithet “pheromone-gland specific” (pgFAR) although specific expression in the pheromone gland is not evident. Phylogenetically, the pgFAR clade containing the genes seems to be specific to Lepidoptera [8]. To date, several genes encoding pgFARs have been characterized in moth species such as *Bombyx mori* [24], *O. nubilalis* [23], *Helicoverpa armigera* [41], and *Spodoptera* spp. [25]. In this study, *CsupFAR2* is confirmed to be involved in sex pheromone biosynthesis of *C. suppressalis*. Previous studies have demonstrated the interplay between the abundance of the pheromone fatty acyl precursors and pgFARs in shaping the pheromone composition [41]. Although *CsupFAR2* shows activity on C_16_–C_18_ fatty acid substrates, it is interesting that *CsupFAR2* shows high selectivity for the Z11-16:acid when both Z11-16:acid and Z9-16:acid are present as substrates (Fig 6d, i). Considering the substrate preference of *CsupFAR2*, the expression of *CsupYPAQ* and *CsupKPSE* in combination with *CsupFAR2* suggested that the FADs and FARs are both engaged and of importance in shaping the ratio of pheromone components in *C. suppressalis*.

Fatty alcohols may be pheromone components for many moth species but often fatty alcohols are transformed to aldehyde pheromone components [27–29]. Since the 1980s, studies about moth aldehyde pheromone biosynthesis are diverging and non-conclusive [27, 42–45]. The enzyme that produces pheromone aldehydes, and thus the gene encoding it, has not been characterized. We heterologously expressed six fatty alcohol oxidases (FAO)/dehydrogenases (ADH)-like genes in *N. benthamiana* leaves but did not get any conclusive results indicating oxidation by any of the candidates. We observed a small amount of Z11-16:Ald in the leaf extracts when Z11-16:OH was produced in large amounts, but this occurred also without heterologous expression of FAO/ADH candidates (Fig. 7). The aldehyde production, in this case, may thus be due to an endogenous FAO/ADH activity from *N. benthamiana*. At the same time, it is interesting to note that all infiltrations of *N. benthamiana* leaves with functional FAD and FAR genes, with or without the acetyltransferase, made the plant release a remarkable amount of Z11-16:Ald as volatiles (Fig. 8c, d). The same infiltrated leaves contained very small amounts of Z11-16:Ald in the leaf extracts (Fig. 8e). This result provides evidence from a plant to support the hypothesis of Foster and Anderson (2019) [44] that in moth females the alcohols and aldehydes are produced and/or stored in different compartments of the gland. In *H. virescens*, they found the aldehydes mainly on the cuticle of the gland whereas the alcohols were found inside the gland beneath the cuticle. In our study, Z11-16:Ald was abundant in the volatiles and less so in the leaf extracts, which implies that the alcohol component Z11-16:OH produced by pgFAR might have been converted to aldehyde when or after it went through the leaf wax cuticle (Fig. 8a).

Acetate pheromone compounds are postulated to be produced by an acetyltransferase from its alcohol precursor. Until now, the genes encoding moth acetyltransferases involved in pheromone biosynthesis have escaped identification. Previously, a plant-derived gene *EaDAcT* that produces 3-acetyl-1,2-diacyl-sn-glycerols (acetyl-TAG) by the acetylation of diacylglycerol and acetyl-CoA [46] was heterologously expressed in *N. benthamiana* for the production of C_14_–C_16_ acetate pheromone compounds with significant but poor activity [5]. Recently, Mateos-Fernández et al. [47] also expressed *EaDAcT* with *Atr∆11* and *HarFAR* in *N. benthamiana*, which resulted in the production and release of Z11-16:OH and Z11-16:OAc. In the present study, the *ATF1* from *S. cerevisiae* which is naturally responsible for the synthesis of volatile esters that contribute to the fruity aroma of fermented alcoholic beverages [48] was co-expressed with moth FADs and a FAR in *N. benthamiana*. It resulted in the production of a high amount of acetate (Fig. 8e) compared to the previous study with *EaDAcT* [5]. This result is consistent with activity differences in the production of acetate pheromone compounds when *ATF1* and *EaDAcT* were expressed in *S. cerevisiae* [30].

Efficient transient expression of insect genes in plant leaves for pheromone production was demonstrated by Ding et al. [5]. Plant-produced pheromones or immediate precursors have been confirmed to perform favorably compared to conventionally chemistry-produced pheromones for trapping male moths [5, 49] and are ready to be introduced for pest management [50]. “Pheromone plants” engineered for the production of insect pheromones may be applied for pest control following at least two different strategies. One strategy is the production of pheromones/pheromone precursors in the plants, isolation, and down-stream processing of the target compounds when the plant tissue has been harvested, for subsequent use of the active ingredient for moth trapping and mating disruption. The other strategy involves developing genetically modified plants capable of releasing pheromone compounds into the environment, and the pheromone-producing plant serves as a dispenser and may directly influence the behavior of insects in the environment.

Optimization of plants as producers and actual dispensers of insect pheromones requires different measures compared to engineering of plants for efficient storage of the same compounds or their precursors. For acetate release, not only a functional acetyltransferase has to be present to produce acetate, but the product has to be released, which makes it important to understand the secretion mechanism of plant volatiles. In a large number of plants, trichomes (tiny specialized hair structures for secondary metabolite production) play a prominent role for release of volatiles. Biosynthesis of plant diterpenes occurs in trichome heads, where secretory vesicles and cells are located [51–53]. We found that the plant carrying *ATF1* controlled by *pCYP71D16*, a trichome-specific promoter cloned from *N. tabacum*, released 3- to 10-fold higher amounts of Z11-16:OAc and also much higher amounts of Z11-16:Ald and Z11-16:OH compared to when expressed from *p35S* (Fig. 8d). This suggests that the release of pheromone compounds benefits from the expression of the pheromone biosynthetic genes in the trichomes. More pheromones might be released along with the native plant volatiles when they are produced in trichomes.

Following several proof-of-concept studies demonstrating the possibility of producing moth pheromones in plants and in microbial cell factories [50], we now show that plants can be engineered to actually release the moth pheromone compounds. This paves the way for the stable transformation of plants required to eventually make use of moth pheromone-releasing plants in different IPM strategies. Development to successful application will depend on the outcome of challenging and exciting behavioral and ecological studies. Sex pheromones are usually mixtures of pheromone components and the behavioral response to a blend of pheromone components depends not only on the presence of components but also on the ratio between the components and their release rates. The behavioral activity of the volatiles released by the genetically modified plants should be assayed by examining different behaviors, attraction, and repellency and mating disruption in specific moth species. Our plants produce a mixture of Z11-16:OAc, Z11-16:Ald, and Z11-16:OH and all three compounds happen to be sex pheromone components of the diamondback moth *Plutella xylostella*. This species would thus be a first suitable candidate for behavioral experiments. Pheromone release from our plants is produced by transient expression of the relevant genes and release rates (amounts) and ratios produced vary from one experiment to the other. Thus obtaining true replicates for a behavioral assay will be a challenge. Electrophysiological experiments to demonstrate the antennal activity of the plant-produced volatiles would be easier to perform but at the same time not really add any conclusive information on “the behavioral activity” of the plants, which is what is of ultimate interest.

## Conclusions

We successfully made *N. benthamiana* release the moth pheromone compounds Z11-16:Ald, Z11-16:OH, and Z11-16:OAc, by transient expression of the functionally characterized *C. suppressalis* ∆11 desaturase *CsupYPAQ*, the fatty acyl reductase *CsupFAR2,* and the yeast acetyltransferase gene *ATF1*. Beyond the aims of our current study, this paves the way for stable transformation and further studies of such pheromone-releasing plants in IPM strategies.

## Methods

### Sequences and phylogenetic analysis

The open reading frames (ORFs) of the FAD-like genes *CsupYPAQ* (GenBank accession number: MN453822) [54] and *CsupKPSE* (MN453823) [55] and the FAR-like gene *CsupFAR2* (MN453825) [56] were obtained from the *C. suppressalis* pheromone gland transcriptome sequences [35]. To identify FAO/ADH-like genes, we retrieved from NCBI sequences corresponding to genes encoding enzymes with similar functions (Table 1). These sequences were then used to query the *C. suppressalis* transcriptome using BLAST [57]. This resulted in the identification of the following gene candidates: *CsupFAO_15570*, *CsupFAO_9572*, *CsupADH_10975*, *CsupADH_14583*, and *CsupADH_17286* (Additional file 1: Table S2)*.* The ORF of the ADH-like gene *HzeaADH7* was obtained from the transcriptome sequences reported by Dou et al. [36, 58]. *CsupYPAQ*, *CsupKPSE*, and *CsupFAR2* correspond to *CsupDes4*, *CsupDes1*, and *CsupFAR2* in [35]. The *AtrΔ11* (JX964774) [59] and *CpuFatB1* (KC675176) [60] were amplified from entry clones [5]. The promoter *pCYP71D16* was cloned from *Nicotiana tabacum* genome as described in Wang et al. [61].

The phylogenies of the FAD and FAR sequences were constructed using *C. suppressalis* FADs and FARs with the functionally characterized sequences retrieved from the GenBank from National Center for Biotechnology Information (NCBI) (https://www.ncbi.nlm.nih.gov/). The Neighbor-Joining tree was constructed using MEGA version 5.0 [62]. The bootstrap consensus tree inferred from 500 replicates was taken to represent the evolutionary history of the analysis of the genes.

### Cloning of gene candidates for functional assay

All the gene candidates were synthesized by Invitrogen, Life Technologies. PCR amplification of each gene candidate was performed using the synthesized sequence as the template with a pair of specific primers (Additional file 1: Table S3) with attB1 and attB2 sites incorporated on a Veriti Thermo Cycler using Phusion Flash High-Fidelity PCR Master Mix (Thermo Scientific ™). Cycling parameters were as follows: an initial denaturing step at 98 °C for 30 s, 38 cycles at 98 °C for 5 s, 55 °C for 10 s, 72 °C for 50 s, followed by a final extension step at 72 °C for 10 min. The PCR products were subjected to agarose gel electrophoresis and purified using the GeneJET Gel Extraction Kit (Thermo Scientific™). Then the ORFs were cloned into the pDONR221 vector in presence of BP clonase (Life Technologies) to generate the entry clone. After the entry clone for each ORF was confirmed by sequencing with M13+ and M13- primers, it was cloned into either the yeast expression vectors pYEX-CHT or pYES52 or the plant expression vector pXZP393 by LR reaction (Invitrogen). The resulting expression clones were analyzed by sequencing.

### Yeast heterologous expression

The experimental workflow is shown in Additional file 1: Fig. S1a. The expression clones containing *CsupYPAQ* and *CsupKPSE* were introduced into the double deficient *ole1/elo1* strain (*MATa elo1::HIS3 ole1::LEU2 ade2 his3 leu2 ura3*) of the yeast *S.c.*, while the expression clones containing *CsupFAR2*, *CsupYPAQ*-*CsupFAR2*, pYEX-CHT empty vector, and pYES52 empty vector were introduced into the *INVSc* strain (*MATa HIS3 LEU2 trp1-289 ura3-52*), using the *S.c.* easy yeast transformation kit (Life technologies). For selection of uracil prototrophs, the transformed yeast was allowed to grow on SC plate containing 0.7% of YNB (w/o aa, with ammonium sulfate) and a complete drop-out medium lacking uracil (Formedium™ LTD, Norwich, England), 2% of glucose, 1% of tergitol (type Nonidet NP-40, Sigma-Aldrich Sweden AB, Stockholm, Sweden), 0.01% of adenine (Sigma). For cultivation of the double deficient *ole1/elo1* strain the medium was supplemented with 0.5 mM methyl (*Z*)-10-heptadecanoate (Z10-17:Me) (Sigma) as an extra fatty acid source.

After 4 days (2 days for *INVSc* strain) incubation at 30 °C, three to five individual colonies were picked independently to inoculate 2 mL selective medium and then grown at 30 °C and 300 r.p.m for 48 h. Yeast cultures were diluted to an OD600 of 0.4 in 5 mL fresh selective medium containing 0.5 mM CuSO_4_ (for pYEX-CHT vector) or 10% galactose (for pYES52 vector) for induction. Then the yeast cells (medium was also harvested for *CsupFAR2* and *CsupYPAQ*-*CsupFAR2* yeast line) were harvested after 48 h incubation in a shaking incubator at 30 °C for fatty acid or alcohol analysis.

### *Nicotiana benthamiana* material and growth condition

The wild type *N. benthamiana* plants for the *Agrobacterium* infiltration were grown in the greenhouse under 16 h/8 h light/dark conditions. The greenhouse's growth temperature and relative humidity were set at 24°C/18°C day/night and 40% RH.

### *Agrobacterium* infiltration of *Nicotiana benthamiana*

The experimental workflow is shown in Additional file 1: Fig. S1b. The genes transformed for pheromone production in plants were generally controlled by the Cauliflower mosaic virus 35S promoter (*p35S*) and Octopine Synthase gene terminator (*tOCS*) but *ATF1* was in one version also controlled by the tobacco trichome-specific promoter *pCYP71D16*. The expression clones containing *CsupYPAQ, CsupKPSE, Atr∆11*, *CpuFatB1*, *ATF1*, *CsupFAR2, CsupFAO_15570*, *CsupFAO_9572*, *CsupADH_10975*, *CsupADH_14583*, *CsupADH_17286* and *HzeaADH7* were introduced into *Agrobacterium tumefaciens* GV3101 strain (MP90RK) by electroporation (1700 V mm^−1^, 5 ms, Eppendorf 2510). A viral silencing suppressor protein *P19* was introduced into GV3101 strain as well in order to inhibit the host cells’ transgene silencing apparatus and extend transgene expression over a longer period of time with a higher degree of expression [63].

The *Agrobacterium* infiltration of *N. benthamiana* started with cultivation of 10 mL of *Agrobacterium* that contained individual gene constructs at 30 °C with LB medium supplemented with appropriate antibiotics overnight in a 300 r.p.m. incubator. Then 100 μM acetosyringone was added to the culture and grown for an additional 2-3 h to induce virulence genes encoded by *Agrobacterium* genome. Subsequently, the *Agrobacterium* were spun down at 4200 g for 5 min at room temperature and resuspended in infiltration buffer (5 mM MgCl_2_, 5 mM 4-morpholineethanesulfonic acid, 100 μM acetosyringone, pH 5.7). Then the optical density under 600 nm wavelength (OD_600nm_) of each *Agrobacterium* culture was measured to adjust the final concentration of each culture to OD_600nm_=0.2, in a total volume of 20 mL infiltration buffer as described before [5].

Afterwards, each final mixture of *Agrobacterium* cells was drawn up into a 1-mL syringe without needle and infiltrated into the underside of a suitable 4-week-old *N. benthamiana* leaf, with a gentle squeeze on the plunger and modest pressure on the leaf using a finger. By this, the *Agrobacterium* solution was forced into the mesophyll spaces wetting the leaf. Five leaves of similar age from three randomly selected 4-week-old individual *N. benthamiana* plants were infiltrated. Then, plants were maintained in the growth chamber for 4 days with sufficient watering. One healthy infiltrated leaf from each plant was analyzed. Two times of independent gene functional assay experiments were performed in this study, each time including three to five replicates.

### Lipid extraction and preparation

For yeast lipid analysis, all the cells and media (for alcohol analysis) were extracted by 1 mL of methanol/chloroform (2:1, v/v), and then 1 mL of water was added to produce a biphasic mixture, which was then vortexed vigorously and centrifuged at 2000 r.p.m for 2 min. Then ca. 330 μL of the chloroform phase containing the total lipids and pheromone compounds was transferred to a new glass vial, followed by evaporation to dryness under a gentle flow of nitrogen. The residues were used for fatty acid and pheromone analysis. For fatty acid analysis, 1 mL of 2% sulfuric acid in methanol was added, and the sample was incubated 1 h at 90 °C for methanolysis. Subsequently, 1 mL water and 1 mL heptane were added and the mixture was vortexed energetically. Finally, ca. 1 mL of heptane phase containing the fatty acids in the form of corresponding methyl esters was transferred to a new glass vial for GC/MS analysis. For pheromone compounds analysis, 200 μL heptane was added after the evaporation to dryness and after vortex vigorously the sample was then transferred to a new GC/MS analytical glass vial.

For leaf lipid analysis, ca. 100 mg of fresh leaf tissue from each sample was collected, and 3.12 μg internal standard methyl nonadecanoate (19:Me) or 1.8 μg internal standard (*Z*)-8-tridecenyl acetate (Z8-13:OAc) was added per gram fresh leaf to the samples for fatty acid and pheromone compound analysis respectively, following the same protocol as described above.

### Sampling volatile compounds in static plant headspace

The setup for plant static headspace volatile collection is shown in Fig. 8b. After 3–5 days of infiltration, the plant was used for the collection of volatiles. The infiltrated plant leaf was enclosed in a glass funnel and covered by a transparent and odorless oven bag, and a solid-phase microextraction (SPME) fiber (65 μm film thickness, polydimethylsiloxane/divinylbenzene (PDMS/DVB), Supelco, Bellefonte, PA) was inserted from the stem of the funnel. The volatiles were collected for 24 h before analysis by gas chromatography/mass spectrometry (GC/MS). The funnel was washed with ethanol and acetone between collections. The collections for each treatment comprised six biological replicates in total. Synthetic Z11-16:OH was used as an external standard to quantify the target compounds.

### Gas chromatography/mass spectrometry (GC/MS)

Yeast and plant-leaf samples were analyzed using an Agilent 5975 mass selective detector coupled to an Agilent 6890 series gas chromatograph equipped with a polar column (HP-INNOWax, 30 m × 0.25 mm, 0.25 μm film thickness) or using an Agilent 5975C mass selective detector coupled to an Agilent 7890A series gas chromatograph equipped with a non-polar column (HP-5MS, 30 m × 0.25 mm, 0.25 μm film thickness). The compounds were identified based on mass spectra and retention times on two columns being identical to synthetic standards. Helium was used as carrier gas (average velocity 33 cm/s). The injector was configured in splitless mode at 250 °C. The oven temperature was set at 80 °C for 1 min, then increased to 230 °C at a rate of 10°C/min and held for 10 min.

DMDS derivatization was performed to determine the position of double bonds in target compounds, according to Dunkelblum et al. [64]. The DMDS-adducts were analyzed by GC/MS equipped with the non-polar column (HP-5MS) under the following oven temperature program: 80 °C for 2 min, then increased at a rate of 15 °C/min to 140 °C, and then increased at a rate of 5 °C /min to 260°C, and held for 3 min.

### Chemicals

Fatty acids references and synthetic pheromone compounds of various origins were available from our laboratory collection.

### Statistical analysis

Data were processed by Prism version 8.0.

## Supplementary Information

**Additional file 1: Table S1**. Expression vectors used for functional assays in this study. **Table S2**. Fatty alcohol oxidase and alcohol dehydrogenase gene sequences. **Table S3**. Primers used in this study. **Figure S1**. Experimental workflow of the heterologous expression in a) yeast and b) plant.

## Data Availability

The datasets used and/or analyzed during the current study are available from the corresponding author on reasonable request. Sequences of the fatty acyl desaturases *CsupYPAQ*, *CsupKPSE* and reductase *CsupFAR2* have been deposited in GenBank (accession numbers MN453822–MN453823, MN453825).

## Abbreviations

16:OH: palmityl alcohol
Z9-16:OH: (Z)-9-hexadecenol
Z11-16:OH: (Z)-11-hexadecenol
Z13-18:OH: (Z)-13-octadecenol
Z9,Z12,Z15-18:OH: linolenyl alcohol
14:Me: Methyl myristate
16:Me: Methyl palmitate
18:Me: Methyl stearate
Z9-14:Me: Methyl (Z)-9-tetradecenoate
Z9-16:Me: Methyl (Z)-9-hexadecanoate
Z11-16:Me: Methyl (Z)-11-hexadecenoate
Z9-18:Me: Methyl (Z)-9-octadecenoate
7,10,13-16:Me: Methyl hexadecatrienoate
Z9,Z12-18:Me: Methyl linoleate
Z9,Z12,Z15-18:Me: Methyl linolenate
16:acid: palmitic acid
Z9-16:acid: (Z)-9-hexadecenoic acid
Z9-18:acid: oleic acid
Z11-16:acid: (Z)-11-hexadecenoic acid
Z13-18:acid: (Z)-13-octadecenoic acid
Z7,Z10,Z13-16:acid: roughanic acid
Z9,Z12-18:acid: linoleic acid
Z9,Z12,Z15-18:acid: α-linolenic acid
IPM: Integrated pest management
FAD: Fatty acyl desaturase
FAR: Fatty acyl reductase
pgFAR: Pheromone-gland specific fatty acyl reductase
ATF: Acetyltransferase
FAO: Fatty alcohol oxidase
ADH: Alcohol dehydrogenase
Z11-16:OAc: (*Z*)-11-hexadecenyl acetate
Z11-16:Ald: (*Z*)-11-hexadecenal
Z13-18:Ald: (*Z*)-13-octadecenal
Z8-13:OAc: (*Z*)-8-tridecenyl acetate
Z9-16:Ald: (*Z*)-9-hexadecenal
Z10-17:Me: Methyl (*Z*)-10-heptadecenoate
19:Me: Methyl nonadecanoate
acetyl-TAG: 3-acetyl-1,2-diacyl-sn-glycerols
GC/MS: Gas chromatography/mass spectrometry
SPME: Solid-phase microextraction
PDMS/DVB: polydimethylsiloxane/divinylbenzene
