# Supplementary material for: Release of moth pheromone compounds from Nicotiana benthamiana upon transient expression of heterologous biosynthetic genes
Source: BMC Biol. 2022 Mar 31;20:80. doi: 10.1186/s12915-022-01281-8 (PMC8969271; doi:10.1186/s12915-022-01281-8)
Supplement: Supplementary file 1 — Additional file 1: Table S1. Expression vectors used for functional assays in this study. Table S2. Fatty alcohol oxidase and alcohol dehydrogenase gene sequences. Table S3. Primers used in this study. Figure S1. Experimental workflow of the heterologous expression in a) yeast and b) plant. [file 12915_2022_1281_MOESM1_ESM.docx]

**Release of moth pheromone compounds from *Nicotiana benthamiana* upon transient expression of heterologous biosynthetic genes**

Yi-Han Xia^1,4^, Bao-Jian Ding^1^, Shuang-Lin Dong^3^, Hong-Lei Wang^1^, Per Hofvander^2^, Christer Löfstedt^1*^

^1^Department of Biology, Lund University, Sölvegatan 37, SE-22362, Lund, Sweden.

^2^Department of Plant Breeding, Swedish University of Agricultural Sciences, P.O. Box 101, SE-23053 Alnarp, Sweden

^3^Education Ministry Key Laboratory of Integrated Management of Crop Diseases and Pests, College of Plant Protection, Nanjing Agricultural University, CN-210095, Nanjing, China.

^4^Department of Biology and Biological Engineering, Chalmers University of Technology, Kemivägen 4, SE-41296, Gothenburg, Sweden.

*Corresponding author: Christer Löfstedt, [christer.lofstedt@biol.lu.se](mailto:christer.lofstedt@biol.lu.se)

**Table S1. Expression vectors used for functional assays in this study.**

| **Genotype and plasmid** | **Heterologous system** |
| --- | --- |
| *MATa elo1::HIS3 ole1::LEU2 ade2 his3 leu2 ura3/*pYEX-CHT | *Saccharomyces cerevisiae* |
| *MATa elo1::HIS3 ole1::LEU2 ade2 his3 leu2 ura3/*pYEX-CHT-CsupYPAQ | *Saccharomyces cerevisiae* |
| *MATa elo1::HIS3 ole1::LEU2 ade2 his3 leu2 ura3/*pYEX-CHT-CsupKPSE | *Saccharomyces cerevisiae* |
| *MATa HIS3 LEU2 trp1-289 ura3-52/*pYES-52 | *Saccharomyces cerevisiae* |
| *MATa HIS3 LEU2 trp1-289 ura3-52/*pYES-52-CsupFAR2 | *Saccharomyces cerevisiae* |
| *MATa HIS3 LEU2 trp1-289 ura3-52/*pYES-52-CsupYPAQ-CsupFAR2 | *Saccharomyces cerevisiae* |
| *Wild-type N. benthamiana/*pXZP393-P19 | *Nicotiana benthamiana* |
| *Wild-type N. benthamiana/*pXZP393-P19-pXZP393-CsupYPAQ | *Nicotiana benthamiana* |
| *Wild-type N. benthamiana/*pXZP393-P19-pXZP393-CsupKPSE | *Nicotiana benthamiana* |
| *Wild-type N. benthamiana/*pXZP393-P19-pXZP393-CsupFAR2 | *Nicotiana benthamiana* |
| *Wild-type N. benthamiana/*pXZP393-P19-pXZP393-CsupYPAQ-pXZP393-CsupFAR2 | *Nicotiana benthamiana* |
| *Wild-type N. benthamiana/*pXZP393-P19-pXZP393-CsupKPSE-pXZP393-CsupFAR2 | *Nicotiana benthamiana* |
| *Wild-type N. benthamiana/*pXZP393-P19-pXZP393-CsupYPAQ-pXZP393-KPSE-pXZP393-CsupFAR2 | *Nicotiana benthamiana* |
| *Wild-type N. benthamiana/*pXZP393-P19-pXZP393-CsupYPAQ-pXZP393-CsupKPSE-pXZP393-CsupFAR2-pXZP393-CsupFAO15570 | *Nicotiana benthamiana* |
| *Wild-type N. benthamiana/*pXZP393-P19-pXZP393-CsupYPAQ-pXZP393-CsupKPSE-pXZP393-CsupFAR2-pXZP393-CsupFAO9572 | *Nicotiana benthamiana* |
| *Wild-type N. benthamiana/*pXZP393-P19-pXZP393-CsupYPAQ-pXZP393-CsupKPSE-pXZP393-CsupFAR2-pXZP393-CsupADH17286 | *Nicotiana benthamiana* |
| *Wild-type N. benthamiana/*pXZP393-P19-pXZP393-CsupYPAQ-pXZP393-CsupKPSE-pXZP393-CsupFAR2-pXZP393-CsupADH14583 | *Nicotiana benthamiana* |
| *Wild-type N. benthamiana/*pXZP393-P19-pXZP393-CsupYPAQ-pXZP393-CsupKPSE-pXZP393-CsupFAR2-pXZP393-CsupADH10975 | *Nicotiana benthamiana* |
| *Wild-type N. benthamiana/*pXZP393-P19-pXZP393-CsupYPAQ- pXZP393-CsupKPSE-pXZP393-CsupFAR2-pXZP393-HzeaADH7 | *Nicotiana benthamiana* |
| *Wild-type N. benthamiana/*pXZP393-P19-pXZP393-CsupFAR2-pXZP393-CsupFAO15570 | *Nicotiana benthamiana* |
| *Wild-type N. benthamiana/*pXZP393-P19-pXZP393-CsupYPAQ- pXZP393-CsupKPSE-pXZP393-CsupFAR2-pXZP393-CsupADH17286-pXZP393-CsupADH14583-pXZP393-CsupADH10975 | *Nicotiana benthamiana* |
| *Wild-type N. benthamiana/*pXZP393-P19-pXZP393-CsupYPAQ- pXZP393-CsupKPSE-pXZP393-CsupFAR2-pXZP393-CsupFAO15570-CsupFAO9572 | *Nicotiana benthamiana* |

**Table S2. Fatty alcohol oxidase and alcohol dehydrogenase gene sequences.**

| Gene name | Sequence |
| --- | --- |
| CsupFAO_15570 | ATGAAGATGCCCGGAAATATGAAACAATATCGCGTGCCGTCGGTGGCGACGGTGCTCTGGATCTTGTTCGTTGCACTGCCGACCGGCACTCAGCAAATCAACCCGATCACATCTTTCATGAACTTCGTCGAAGAAGGCACAAGGCAGCTCGACGATGAACCACCGGATCAAGCGCGTCTGCTCACCGAATATGATTTCATCGTCGTAGGAGCAGGGACAGCCGGGTGTGTCATAGCGAACCGTTTAACAGAAGTACCGGATTGGAAAGTGTTATTAATTGAAGCTGGTGTCAACGAAAATTATGTCATGGACATCCCACTTGTTGCCAACTACCTCCAATTTACTGAAGCCAATTGGAAGTATAAGACACAACCTTCGAACAGATACTGTGCTGGTTTTGATAACAAGCAATGCCACTGGCCACGCGGAAAAGTTGTCGGAGGATCCAGTGTCTTGAACTACATGATATACACCAGAGGCTCTCAACCCGATTACGATAACTGGAGGGGCATGGGCAACGAAGGTTGGGGCTGGGATGAAGTCTTACCCTATTTCAAGAAGATCGAAAATTTTCAAATTCCTTCATTCAATAATTCTAAATATCATGGAAATAAGGGCTACCTCAATGTCGAACATGCTCCGTTTCGAACACCAGTAAGCAAAGCATGGGTTAAAGGCGCACAACAACTCGGTTTCAAGTATGGAGATCATAACGGCGAGAACCCAGCAAGTGTATCATTTTTACAACTCTCAATGAAAAATGGAACACGACACAGCTCTAGTCGGGCTTACTTACACCCAATAAATGGCAGAAAAAATCTTCATGTTTCCAAAGCTAGTATGGTCACGAAGCTGATTTTTGACGAGACCAAAACCAAAGTACTAGGTGTGGAACTGGAGAAACATGGCATGAAATACAAAATATTAGCAAATAAAGAAGTCATTCTATCTGCGGGTGCATTAAATACCCCTCAAATACTGATGCTATCCGGTATAGGGCCGAAGCAACATTTAGATTCATTGAAAATCGAGGTGGTTAAAGATCTGCCAGTCGGGTACAACTTGATGGATCATATAGCAGCAGGAGGCCTACAATTCATTGTGCGGCCACAAAACTTAAGTTTGAGCACTCGATATGTTTTGAACCACCTAGATATTGTTTTCAAATGGATGAAGACCCACAAGGGACCATTGTCAGTGCCTGGTGGATGCGAAGCTCTTGTTTTTACAGATTTAAAAGACAAATTCAATCCAAAAGCCTGGCCTGATATGGAATTGCTCTTTATTGGTTCTAGTTTAAATGCAGATCCCTTGCTGCAATACAACTTCAATTTCGACAAAAATATATACAGTGACACATTCGGACCTATGGGAAACGCAGATGCTTTTATGGTATTCCCAATGCTGATGCGGCCAAAGTCTAAAGGAAGAGTAATGTTACAAAGTAGAAATCCAAAAGCACCACCAATACTAATACCAAACTACTTTGAATATGAGGAAGACTTGAAGAAGATTGTGGAAGGAATGAAACTGGCGGTACAGATTTCAAGACAGCCTGCTATGAGAGCGATAGGAGCGAAATTGTATGACGTACCGATTGAGGACTGTTTGAAGTACGGGCCATTTGGGAGTGACGAGTACTTCGCTTGCCATGCTCAGATGTTTACTTTTACGATATACCATCAGAGCGGAACGTGTAAGATGGGTGTCGCCGACGATCCCTCTTCTGTAATCGATTCAAGATTACGAGTACATGGTATCGAAAGGTTGCGAGTGATAGATGCTAGTGTAATGCCTGAAATAGTTGCAAGTCATACAAATGCTCCAGTGTATATGATAGCGGAGAAAGGATCGGATATCATAAAAGAGGATTGGAACAGATTTTATACCATTTAG |
| CsupFAO_9572 | ATGCAGTGGATCCTGCTGTCCCTGTACATTGCTATGGCTAACGCTATGGAAACGACCAACCTGTTCGACTTCTGGACCGACCTGTTCCGTCCTCTGCCTCGTAACCCTCGTGAAGGTTTCATGTCCGACTACACCCCTATCGACCAAGAGGAATTCGACTTCATCATCGTCGGTGCTGGTTCCGCTGGTTGCGTGCTGGCTAACCGTCTGACTGAGATCCCCGAGTGGAAGATCCTGCTCATCGAGGCTGGTGGCAACGAGAACTTCTTCTCTGACATCCCTATCTTCGCTGCCTTCCTGTCCACCACTCCTATGAACTGGATGTACAACTCCGAGCCTGAGCAGAAGGCTTGCAGGGACTTGCGTGGCAACGTGTGCTTCCTGCCTCGTGGAAAGGTGCTCGGTGGTTCCTCCGTGCTGAACTTCCTGATCTACCAGCGTGGTCACCCCGACGACTACGACGACTGGGCTAAGATGGGAAACCACGGCTGGTCCTACTCCGAGGTGCTGCCCTACTTCAAGAAGTCCGAGAACATCAAGATCTCCAGCCTGCGCAACTCCACCTACCACGGTCGTGGTGGTTACCTGGACATCGAGTACGCTCCCTACAAGTCCCCTCTGGAACGTCTGTTCAAACGTGCTGGCGAGGAACTGGGCTACGAGTGGCGTGATCCTAACGGCGAGCAAGTGATCGGTTTCTCCAAGCCTCAGGCTACCATGCGTAACGGTCGTCGTTGCTCTACCTCCAAGGCTTTCCTCGAGCCTATCCGTTTCCGTCGTAACCTGAAGGTGTCCAAGCACTCCATGGTCAACAAGATCCTGATCGACCCTCGTACCAAGACCTCCTACGGTGTCGAGCTGACCAAGCAGATGAAGCGTATCCGTGTGCGTGCTCTGCGTGAAGTGATCCTGGCTGCTGGTTCTATCGGTTCCGCTCAGCTGCTGATGGTGTCCGGTGTCGGTCCTGAAGAACACCTCCGCGAGATGGAAATCGAGCCCGTGGTTAACCTGCCTGTGGGTTACAACCTGCAGGACCACGTGACCTTCTCCGGCAACGCTTTCATCGTGAACGACTCTTCCCTGACCGTCAACGACATGCTGGCTGCTTCACCCCTGTCCGCTATCGCTTACTTGGCTGGTCGTGGTCCCCTGACTTTGCCTGGTGGTGCTGCTGGACTGGCTTTCACCCGTTCACCTTACGCCATGGACGACCACTCACCTACTCGTCCCGACATCGAGCTGGTCATGGGTGGTGGTTCTCTGGCTGGCGACCTGTTGGGTATCCTGCGTTCTCTGCTGGGTGTCACCGACCAGTGGTACTACCGTATGTACTCCTCTCTGCCCATGCGTGTGCGTCAGAACACCTTCTCTATCAACCCCGTGCTGATCCGTCCTCGTTCCATCGGTCGTCTGAAGCTGCGTTCCGGCAACTTCTCTGATCACCCCTCCATCAAGATGAACTACTTCGACGACCCCAAGGACCTGAAGGCTCTGGTCAACGGTGTCCGTCTGATCCAGCAAGTCATCGGCACCTCCGCTTTCCAGCAGTACGAGACTAGACTGCACGACGTGCCATTCCCTGGCTGCGAGAACGTGTTGTTCGACTCCGACCAATACTGGGAGTGCGCTATCATGCAGACCGCTATCACCCTGGACCACCAAGTGGGAACCTGCAAGATGACTCCCGCTGGCGATCCTTCTGGTGTCGTGTCTCCTCGTCTGCTGATCCACGGAATGCGTGGACTGAGAGTGGCTGACGCTTCTATCATCCCTCGTATCCCCGCTGCTCACACCAACGCTCCCGTCATCATGATCGCTGAGAAGGCTGCTGACCTGATCAAAGAGGACTGGGGCATGCACCGTGTGACCACCAACTTCATCGACGGTTCTTAA |
| CsupADH_10975 | ATGTCCGAGGACAACCTGACCGCTATGCTGTACAAGACCAAGGACCTGCGTCTGGTGCAGACCCCTATTCCTGAGATCGCTGAGGACGAGGTGCTGCTGCGTATGGACTGCGTTGGTATCTGCGGTTCCGACGTGCACTACTGGAAGTCCGGTTCCTGCGGTCCCTTCATCGTGAAAGAACCTATGATCATGGGTCACGAGGCTTCCGGTGTCGTGGCTAAGCTGGGTGCTAAAGTGAAGTCCCTGAAAGTGGGCGACCGTGTGGCTATCGAACCTGGTGTTCCTTGCCGTTACTGCGAGTTCTGCAAGACCGGTCGTTACCACTTGTGCCCCGACATCGTGTTCTGCGCTACTCCTCCTTGCCACGGCAACCTCGTGCGCTACTACAAGCACGCTGCTGATTTCTGCTACAAGCTGCCCGACCACGTGACCATGGAAGAGGGTGCTCTGCTGGAACCTCTGTCCGTGGCTGTTCACGCTTGTCGTAGAGCTGCTCTCGGTCCTGGTCAATCCGTGTTGGTTCTCGGTGCTGGCCCTATCGGACTGCTGCTGATGTTGACCGCTCGTGCTATGGGCGCTAACAAGATCCTGATCACCGACATCCTCGAGTCCCGTCTGGAATTCGCTAAGAAGCTGGGCGCTGACGCTACCCTGTTGGTGTCATCTGCTGACTGCGAGGCTGAGCTGGTGGCTAGAGTGCACGACCTGTTGGGTTGTCACCCCGACGTGTCCTTCGACGCTTCTGGTGCTAACGCTTCCGTGCGTCTGGCTCTGTTGGCTACCAAGTCTGGTGGTGTCGCTGTGCTCGTCGGAATGGGTGGTCCTGAACAGACTGTGCCTCTGGGTGCTGCTCTGACCAGAGAAGTGGACGTGCGCGGTATCTTCCGTTACGTGAACGAGTACCCTATCGCTCTGAACATGGTGGCTAACGGTCACATCAACGTGAAGCCCCTGGTCACCCACCACTTCGACATGGAACAGACCGTCGAGGCTTACAACACCACTCTGAAAGGCCAGGGCATCAAAGTGATGATCCACGTGCAGCCCCGTGACACTAACAACCCCGTGAAGTTCTAA |
| CsupADH_14583 | ATGAGTACAGTTGGAAAAGTGATAAAATGTTTAGCAGCCGTTGCCTGGGAAGCCGGCAAGCCATTATCAATTGAAGAGATCGAAGTGGACCCACCCAAAGCCGGCGAAGTCCGCGTACAGATCACTGCTACCGGCGTATGTCACACTGACGCATATACTTTGTCCGGTAAAGACCCTGAAGGAGTATTTCCAGTAGTTTTGGGCCATGAGGGTGGTGGTATCGTAGAGAGCGTCGGTGAAGGAGTAACATCCGTGAAACCAGGTGATCACGTCGTTCCACTCTACGTTCCGCAATGTAAAACATGCAAATTCTGTTTGAACTCCAAAACCAATCTATGCCAGAAAGTGAGAGTTACACAAGGACAAGGTGTAATGCCCGATGGTACTAAGCGATTCCGTTGTAAAGGTCAAGAATTATACCATTTCATGGGATGTTCAACCTTCAGTCAGTACACAGTAGTTTTAGAAATTTCTATTTGCAAAGTGAATGAAGCCGCTCCATTGGATAAAGTCTGTCTATTGGGCTGTGGAATCCCTACTGGTTATGGAGCAGCTCTGAATACAGCTAAGGTGGAACCTGGTTCCAATTGTGCAATCTTTGGTCTTGGAGCAGTCGGTCTGGCAGTAGCTCTTGGATGTAGAGCAGCTGGTGCAAAACGTGTCATAGGAGTGGATATTAATCCTGCTAAGTTCGAGGTAGCCAAAAAGTTTGGTGTCACCGAGTTTGTCAACCCTAAGGATTATGACAAACCCATTCAAGAAGTATTGGTGGGTTTGACAGATGGAGGATTAGACTACACATTTGAATGCATTGGTAATGTGAACACCATGCGATCTGCTCTGGAAGCTTGTCATAAGGGTTGGGGAGTTTCAGTTATCATTGGAGTAGCTGGTGCTGGTGAAGAGATCAGCACTCGTCCCTTCCAGCTGGTGACTGGTCGCACCTGGAAGGGAACTGCTTTTGGAGGTTACAAGAGCCGTGACAGTGTACCCCAGCTGGTTGATGAATATCTTTCTAAGAAACTGCCTATTGATGATTTTGTGACGCACAATGTGTCATTAAAGGAGATAAATGAAGCATTTCATCTAATGCATTCGGGCCAGTCTATTCGTGCTGTTGTACATTTTTGA |
| CsupADH_17286 | ATGGCTGAGTCAAATTCAACAAACTCAGAAGACAAAAGTAAAGTACCGTCGGTGATGAGAGCAGCACAGCAGACCGGATTTGGAAATGTTCGTGAGGTACTGAAGCTAAGTGATGATGTGCATGTTCCATATGAGCTAGAGCCCAAGCAAGTTCTCGTTCAGGTCTATGCTGCCTCAATTAATCCAATTGACTGGAAGACATTGAATGGGAACCTATCCCTTATCACACGGTTCTCGTTTCCACATATCCCTGGAAAGGATGTTGCTGGGATTGTTGTTGCTACAGGTTTACATGCTGAGCGCTTTTGTATTGGAGACGGGGTTTATGGAAATCTGGGTATGAGTGATGGCAGCTATGCCGAGTATGTTCGTGTTGATGAGTCATTACTTTCCAAGAAACCAAATAATATAACAATGGAAGAAGCAGCAGCAATCCCTCTAGCCTGTGGAACAAGCTACCAAGCCTTGTTTAATAAGGTCTCTCCGCCACTTGGGAGACAAAGTAAAATACTAATTCTGGGCGGAAGCACTGCAACGGGCCTATATGCACTCCAATTGGCTAAAGCGACCGGAGCATTTGTTACGGTGACATCTTCGCAGCGAAATTTTAATCTTCTGGAAAGTCTTGGGTTTAGTCTTGTGCATGGAAAACCCAACCCGGATGCTGATGAAAAGCAGCTTCATGTGATTGATTATAATGAAAAGGATTTTGGAAGGGAGTTAAAAGGCGAAGACTATGACGTTGTTTATGACTGTGTTGGTGGCAGACAGCAATGGGTGTCTGCAAAGCAGGTACTAAAGCGCAATGGGCAGTTTATCACAATTGTCGGCGATGACCCAAAAACAGTTCTATCTCTGAAGTCAATAATTTCATTTAGTTCCAAATTGATAGGTCGAAAGTTTCTGTCATTCTTTGGCTGCTCGCACTATAATTATGTCTTCCATACTCATGAGCCCAAATCTTCAGATCTTGATGAAATTAGAACGAAGTATATTGAAACAAAAAAAGTTAGGCCATTAATCGACACGATTTTTGACTGGAAAAAGGATGGCATTGAGGCACTATACTCGCTATATGAGAAATCAAAGAGTGGTAAAGCACAAGGAAAGCTAGTTCTTAGAATAGCCGATTAG |

**Table S3. Primers used in this study.**

| Gene abbreviation | Primer sequence (5’-3’) |
| --- | --- |
| *CsupYPAQ_F*  *CsupYPAQ_TT_79R*  *TT_145R*  *TT_attB5r_R*  *attB5_Gal1_ F*  *Gal1_CsupFAR2_F*  *Gal1_CsupFAR2_R*  *CsupFAR2_404F*  *CsupFAR2_1257R*  *Gal1_94F*  *Gal1_395R*  *CsupELO1_F*  *CsupELO1_R*  *CsupELO3_F*  *CsupELO3_R*  *CsupELO4_88F*  *CsupELO4_783R*  *Csup15570_605F*  *Csup15570_1550R*  *Csup9572_553F*  *Csup9572_948R*  *Csup10975_295F*  *Csup10975_625R*  *Csup14583_170F*  *Csup14583_633R*  *Csup17286_307F*  *Csup17286_1097R*  *HzeaADH7_37F*  *HzeaADH7_R* | GGGACAAGTTTGTACAAAAAAGCAGGCTTAATGGCCCCGAATTCAATTCAAAATG  CTAACATAACTATAAAAAAATAAATAGGGACCTAGACTTCAGGTTGTCTAACTCCTTCCTTTTCGGTTAGAGCGGATTCAATCCTCCGTCTTGCTGTAC  GTTACATGCGTACACGCGTCTGTACAGAAAAAAAAGAAAAATTTGAAATATAAATAACGTTCTTAATACTAACATAACTATAAAAAAATAAATAG  GGGGACAACTTTTGTATACAAAGTTGACTCTTCGAGCGTCCCAAAACCTTCTCAAGCAAGGTTTTCAGTATAATGTTACATGCGTACACGCGTCTG  GGGGACAACTTTGTATACAAAAGTTGTAACGGATTAGAAGCCGCCGAGCGGGTGAC  ACGTCAAGGAGAAAAAACCGTGGTTGTGAAAATGGAAC  GTTCCATTTTCACAACCACGGTTTTTTCTCCTTGACGT  CTCTGTCCGAGGCTATCATCATC  CTTGTCGATGAACAAGAACTGGTTG  GATGTGCCTCGCGCCGCACTGC  GAGGTATATTAACAATTTTTTGTTGATAC  GGGGACAAGTTTGTACAAAAAAGCAGGCTAATGGAGGTGCTAAGGAGACTAG  GGGGACCACTTTGTACAAGAAAGCTGGGTTTTACTGGGACGCCACCGCTCCTGCCATC  GGGGACAAGTTTGTACAAAAAAGCAGGCTAATGAACGGTGCTAATACGACCTTCGAAATGTC  GGGGACCACTTTGTACAAGAAAGCTGGGTTCTAGCAATCTTTTGCTTTTCCATTGGCTG  CTGATCATCTGCCTGTCCTACGTG  CTTAGCGCGCACTTTGGTCTTGG  GATCGAAAATTTTCAAATTCCTTCATTC  CAAGTCTTCCTCATATTCAAAGTAG  AGGAATTCGACTTATCACGG  CATCAACAATTGGGCAGATC  GCTAAAGTGAAGTCCCTGAA  TGATCAGGATCTTGTTAGCG  GTCACACTGACGCATATACT  ACCAAAGATTGCACAATTGG  GTTGCTACAGGTTTACATGC  TATAGTGCCTCAATGCCATC  GAACCACACCAAGAAGATCT  ATCACGTAGTTCTCGTTAGC |

**Figure S1. Experimental workflow of the heterologous expression in a) yeast, and b) plant. Adapted from Löfstedt and Xia [49].**
